# Dual-Path Adversarial Denoising Network Based on UNet

**DOI:** 10.3390/s25154751

**Published:** 2025-08-01

**Authors:** Jinchi Yu, Yu Zhou, Mingchen Sun, Dadong Wang

**Affiliations:** 1 School of Mathematics and Computer Science, Jilin Normal University, Siping 136000, China; yyu@mails.jlnu.edu.cn (J.Y.); zzhou@mails.jlnu.edu.cn (Y.Z.); 2 School of Computer Science and Technology, Jilin University, Changchun 130012, China; mcsun20@mails.jlu.edu.cn; 3 Jilin Provincial Key Laboratory for Numerical Simulation, Jilin Normal University, Siping 136000, China

**Keywords:** image denoising, three-module architecture, dual UNet, adversarial training

## Abstract

Digital image quality is crucial for reliable analysis in applications such as medical imaging, satellite remote sensing, and video surveillance. However, traditional denoising methods struggle to balance noise removal with detail preservation and lack adaptability to various types of noise. We propose a novel three-module architecture for image denoising, comprising a generator, a dual-path-UNet-based denoiser, and a discriminator. The generator creates synthetic noise patterns to augment training data, while the dual-path-UNet denoiser uses multiple receptive field modules to preserve fine details and dense feature fusion to maintain global structural integrity. The discriminator provides adversarial feedback to enhance denoising performance. This dual-path adversarial training mechanism addresses the limitations of traditional methods by simultaneously capturing both local details and global structures. Experiments on the SIDD, DND, and PolyU datasets demonstrate superior performance. We compare our architecture with the latest state-of-the-art GAN variants through comprehensive qualitative and quantitative evaluations. These results confirm the effectiveness of noise removal with minimal loss of critical image details. The proposed architecture enhances image denoising capabilities in complex noise scenarios, providing a robust solution for applications that require high image fidelity. By enhancing adaptability to various types of noise while maintaining structural integrity, this method provides a versatile tool for image processing tasks that require preserving detail.

## 1. Introduction

Images are crucial for the dissemination of information and are widely used in various fields, including scientific research, industry, healthcare, security, and daily life. Separating noise from images while preserving the clean image is a vital preprocessing step in many visual tasks, including image classification, image segmentation, medical imaging, image compression, video denoising, and object detection in remote sensing imaging. Image quality has a direct impact on the accuracy and efficiency of subsequent analysis, recognition, and decision-making tasks. Therefore, it is of great significance to enhance the image quality by restoring the original details and clarity through a model.

Traditional denoising methods, while effective in specific scenarios, have obvious limitations in handling complex noise patterns, preserving details, and adapting to various types of noise [1,2,3,4,5]. For example, wavelet transform-based methods may cause artifacts when processing high-frequency components. Non-local means (NLMs) methods [6], though leveraging image self-similarity, suffer from low computational efficiency and performance degradation in high-noise scenarios. Even recent improvements have not solved these problems [7]. Block-Matching and 3D Filtering (BM3D) [8] enhances denoising performance, but it faces scalability issues with large datasets. Focusing primarily on local information, these methods struggle to capture global image structures and long-range dependencies, leading to unsatisfactory results in complex situations. For instance, some techniques struggle to balance noise removal and detail preservation in scenarios involving Gaussian noise. When removing salt-and-pepper noise, it often causes over-smoothing and texture loss. These shortcomings underscore the need for a more robust and comprehensive denoising framework [9].

Convolutional Neural Networks (CNNs) and Generative Adversarial Networks (GANs) have demonstrated significant potential in image processing tasks. CNNs excel at learning hierarchical feature representations, effectively removing noise and restoring details. GANs enrich denoising models’ training data by generating realistic noisy images through adversarial training. These technologies show significant promise in handling complex noise patterns and preserving key texture information. Researchers have proposed at least dozens of CNN-based denoising methods for general or specific images [5]. For example, DnCNN uses deep convolutional networks for efficient denoising [10]. Noise2Noise expands the applicability of denoising models by utilizing unsupervised training with noisy image pairs [11], thereby eliminating the need for clean reference images. These CNN-based denoising methods consistently outperform traditional denoising techniques. However, CNN-based denoising networks depend heavily on extensive and challenging-to-create training datasets. Additionally, the performance of these models can be significantly affected by discrepancies between the simulated noise and the actual noise. GAN-based methods such as ID-MSE-WGAN [12] and Trident GAN [13] can also lead to specific improvements. The training of GANs is also typically complex as the generator and discriminator struggle to reach an equilibrium, leading to convergence difficulties and generation quality that is difficult to control.

In light of the limitations of the aforementioned denoising methods, we propose a novel image-denoising framework designed to address these issues. The framework combines a UNet-like encoder–decoder architecture with adversarial training. The framework adopts a dual-path adversarial mechanism. The generator synthesizes diverse noisy images to enhance the training data. Meanwhile, the denoiser, with a dual-UNet structure, extracts multi-scale features and fuses dense features to strengthen noise removal and detail restoration. The discriminator assesses the authenticity of the generated images and optimizes the denoising process. This adversarial setup not only enhances the model’s adaptability to various noise types but also boosts its robustness in complex scenarios, offering a comprehensive solution to the challenges faced by traditional methods.

The contributions of this paper can be summarized as follows:

- We design a dual-path adversarial structure that takes a set of clean and noisy images as input during training. The model can continuously optimize itself through the adversarial structure, thereby enhancing the denoising performance.
- We modify the UNet architecture and propose a new dual-U-shaped network model. This model can extract image features more finely and effectively reduce image noise.
- We also verify other multi-U-shaped network designs through experiments, demonstrating the practical reliability of the proposed dual-U-shaped network.

## 2. Related Work

In the field of image denoising, researchers are constantly exploring. The U-shaped encoder–decoder structure is a typical application of fully convolutional networks in image denoising. It uses downsampling layers to reduce image spatial resolution and mine semantic content and structural information. Through skip connections, it preserves image details and edge information, which is of great significance for maintaining feature continuity and improving denoising accuracy. The UNet network has shown outstanding performance in biomedical image segmentation and other fields [14]. The UNet++ architecture further improves performance by nesting UNet structures and enhancing skip connections [15]. MS-UNet-v2 performs well in medical image segmentation tasks using small training datasets through adaptive denoising methods and other techniques [16]. UNet-like architectures are susceptible to input resolution. When the spatial size of test images deviates significantly from that of the training crops, skip connections may fail due to feature map misalignment, resulting in edge artifacts. Our Dual-UNet mitigates this issue by employing multi-scale random cropping and online re-sampling during training and by inserting a deformable alignment module at every skip connection. This module automatically registers feature maps in both spatial and channel dimensions, ensuring that high-resolution test images fully exploit cross-layer details. But excessive downsampling can lead to blurred image details. Therefore, it is crucial to reduce the number of downsampling layers while maintaining tight connections between layers. Our proposed dual-UNet structure significantly improves denoising performance.

Skip connections were initially used to address the vanishing and exploding gradient problems in neural network training. As network depth increases, training efficiency declines. Adding skip connections enables the propagation of low-level learned weights to higher layers, allowing for efficient feature capture, accelerated training, and improved performance [17]. The DenseUNet model achieves remarkable results in image denoising tasks through dense skip connections and feature fusion [18]. Although dense connectivity alleviates gradient vanishing, it causes the number of feature channels to grow exponentially with depth, leading to a quadratic increase in GPU memory consumption. The dual UNet addresses this by replacing traditional dense connections with a combination of serial and parallel approaches. It retains only lightweight residual blocks within each sub-network, which helps keep the overall memory usage comparable to that of a single UNet. Additionally, skip connections can expand the receptive field of the network. Our designed MRDB blocks reduce the network’s reliance on isolated learned features, better utilizing skip connections. Dense skip connections continuously introduce features from different levels. After undergoing feature fusion, the model can capture multi-level image features, laying the foundation for generating clear images.

Dilated convolution controls the receptive field of the convolution kernel by adjusting the dilation rate [19], enabling the model to capture more contextual information. In image denoising, it can enlarge the receptive field to capture low-frequency structural information better and suppress high-frequency noise. Meanwhile, it maintains the feature map resolution, which is crucial for preserving image details. In image denoising applications, dilated convolution is often combined with other deep learning techniques, such as CNN and ResNets, to build efficient denoising models [20,21]. Chen et al. studied the application of dilated convolution in semantic image segmentation [22]. The multi-scale denoising network based on dilated convolution proposed by Wang et al. improved denoising results [23]. The multi-scale dilated convolutional neural network by Li et al. also effectively enhanced denoising performance [24]. Cascaded dilated convolutions are prone to “gridding artifacts,” manifesting as checkerboard patterns in smooth regions. Moreover, excessively large dilation rates cause the effective sampling grid to become overly sparse, resulting in the loss of fine-grained texture details. The MRDB block we designed uses dilated convolutions with different parameter-adjusted sizes. It implicitly uses downsampling to generate multi-scale features, adaptively suppressing artifacts generated by overly large dilation rates, which is beneficial for learning image contexts. Experiments have shown that denoising models utilizing dilated convolution can achieve effective noise reduction while preserving image details and textures.

Generative Adversarial Networks (GANs) were proposed by Ian Goodfellow and his team in 2014 [25]. These networks generate realistic data samples through adversarial training between a generator and a discriminator. In image denoising, GANs are widely used to learn the mapping between noisy and clean images, thereby removing noise. The generator creates noisy images, and the discriminator determines whether an input image is a real-world noisy image or a generated one. Through adversarial training, the generator learns to produce more realistic noisy images, indirectly learning to recover clean image information from noisy images. Isola et al. explored conditional adversarial networks for image-to-image translation tasks [26]. Lai et al. combined GANs with Laplacian pyramid networks for image super-resolution tasks [27]. Arjovsky et al. proposed a GAN training method based on the Wasserstein distance to alleviate the mode-collapse problem [28]. Thus, GANs show great potential in image denoising. In addition, Tai et al. designed MemNet to remove image blur effectively [29]. Wang et al. proposed the LG-BPN network, which utilized a low-resolution guided bidirectional pyramid structure to enhance image denoising performance [30]. Wu et al. constructed a dual residual attention network, combining residual learning and attention mechanisms to boost image denoising performance [31]. These approaches can effectively remove image noise while preserving details and texture information. However, training GANs is extremely sensitive to hyperparameters; even minor adjustments in learning rates or loss weights can trigger mode collapse or training oscillations. In addition, adversarial losses often sacrifice pixel-level fidelity in favor of perceptual realism, which can introduce structurally unacceptable errors in high-fidelity domains, such as medical imaging. To address these issues, we adopt a composite loss: the adversarial loss is used solely for distribution alignment, the pixel-wise loss enforces strict per-pixel fidelity, and a feature-level loss locks structural consistency. Gradient penalty and early stopping are further introduced to stabilize convergence and simultaneously preserve both perceptual quality and numerical accuracy. Meanwhile, we utilize a specially designed denoising model to ensure compatibility with the generator, stabilize image quality, and employ generated noisy images to assist in denoiser training, thereby enhancing the ability to process noisy images.

## 3. Network Structure

### 3.1. Overall Structure

**Figure 1:**
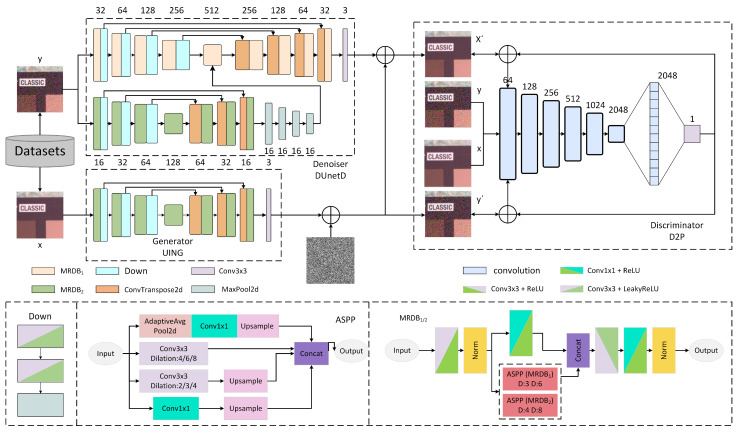
Overall network structure.

We propose a denoising model that combines a dual-path UNet structure with a GAN. As shown in Figure 1, the overall network processes images by inputting noisy and clean images into the discriminator, a clean image into the generator and a noisy image into the denoiser, for dual-path adversarial training. The generator learns to produce realistic-looking noisy images paired with real, clean ones as fake samples. The denoiser is trained to remove noise from real noisy images, with the processed images paired with the original noisy ones as another set of fake samples, yielding real clean and corresponding noisy images from real samples. These three sets of images are input into the discriminator for joint distribution discrimination. Through continuous adversarial learning, the generator gets better at generating realistic-looking noisy images, thereby indirectly learning to recover clean images from noisy ones. This mechanism enables GANs to excel in image denoising, effectively removing noise while preserving details and textures. Algorithm 1 illustrates the running process of the model we designed.
**Algorithm 1** Dual-path adversarial denoising network training procedure**Input**:Clean image set $X=\{x_{1},\ldots ,x_{n}\}$.Noisy image set $Y=\{y_{1},\ldots ,y_{n}\}$ (unpaired).Hyper-parameters: α, $\tau_{D}$, $\tau_{G}$, $\lambda_{\mathrm{gp}}$, ksize, batch_size, epochs, early_stop_patience **Parameters**:$\theta_{D}$ — Denoiser (Dual-UNet) parameters.$\theta_{G}$ — Generator (UNet-G) parameters.$\theta_{P}$ — Discriminator (PatchGAN) parameters.**Output**:
Trained denoiser $D(x^{\prime };\theta_{D})$ with minimal noise and maximum detail preservation1:Randomly initialize $\theta_{D}$, $\theta_{G}$, $\theta_{P}$2:Pre-compute Gaussian kernel K=get_gausskernel(ksize,C=3)3:**for** epoch =1 **to** epochs **do**4:    **for** mini-batch =1**to** ⌈|X|/batch_size⌉ **do**5:        /* 1. Sample data */6:        {x}← Random batch from *X*7:        {y}← Random batch from *Y* (same size)8:        {z}←N(0,1) noise map same spatial size as *x*9:        /* 2. Forward passes */10:        $\hat{x}\leftarrow y-D\left(y;\theta_{D}\right)$                                                                     ▹ Denoised image11:        $\tilde{y}\leftarrow G\left(\mathrm{concat}\left(x,z\right);\theta_{G}\right)$                                                          ▹ Synthetic noisy image12:        /* 3. Update Discriminator *P* */13:        $L_{P}\leftarrow \alpha \left(P\left(\hat{x},y;\theta_{P}\right)-P\left(x,y;\theta_{P}\right)\right)+\left(1-\alpha \right)\left(P\left(x,\tilde{y};\theta_{P}\right)-P\left(x,y;\theta_{P}\right)\right)+\lambda_{\mathrm{gp}}\cdot \mathrm{gradient\_penalty}$14:        $\theta_{P}\leftarrow \theta_{P}-\eta_{P}\nabla_{\theta_{P}}L_{P}$15:        **if** mini-batch mod num_critic =0 **then**16:           /* 4. Update Denoiser *D* */17:           $L_{D}\leftarrow -\left(1-\alpha \right)P\left(\hat{x},y;\theta_{P}\right)+\tau_{D}{∥\hat{x}-x∥}_{1}$18:           $\theta_{D}\leftarrow \theta_{D}-\eta_{D}\nabla_{\theta_{D}}L_{D}$19:           /* 5. Update Generator *G* */20:           $L_{G}\leftarrow -\alpha P\left(x,\tilde{y};\theta_{P}\right)+\tau_{G}{∥\mathrm{GF}\left(\tilde{y}-x\right)-\mathrm{GF}\left(y-x\right)∥}_{1}$21:           $\theta_{G}\leftarrow \theta_{G}-\eta_{G}\nabla_{\theta_{G}}L_{G}$22:        **end if**23:    **end for**24:    /* 6. Early stopping check */25:    val_loss ← MAE on validation set26:    **if** val_loss has not improved for early_stop_patience epochs **then**27:        break28:    **end if**29:**end for**30:**return** $D(x^{\prime };\theta_{D})$

To overcome the inherent trade-off between global structure preservation and local detail recovery that plagues traditional single-path denoising networks, we propose a dual-path adversarial architecture. In the first path, a generator learns to “forge” noise by mapping clean images to realistic noise distributions that can deceive the discriminator into regarding them as authentic noisy samples. In the second path, a denoiser learns to “restore” the truth by reverting authentic noisy images to their clean counterparts, rendering them indistinguishable from genuine clean images to the discriminator. Both paths converge at the shared discriminator, establishing a dynamic adversarial interplay: the generator progressively refines the accuracy of noise simulation, thereby supplying the denoiser with richer and more challenging training samples, while the denoiser, guided by adversarial feedback, continually enhances its capacity for preserving edges and textures. By sharing a single discriminator, the system achieves closed-loop optimization of “noise synthesis–noise removal,” thereby expanding the model’s adaptability to diverse noise distributions and mitigating the overfitting risks inherent in fixed-prior single-path designs. We adopt the UNet structure because it already has excellent performance. We utilize a dual-UNet to optimize the efficiency of the UNet architecture.

### 3.2. Denoiser Double-Layer U-Denoise Network

**Figure 2:**
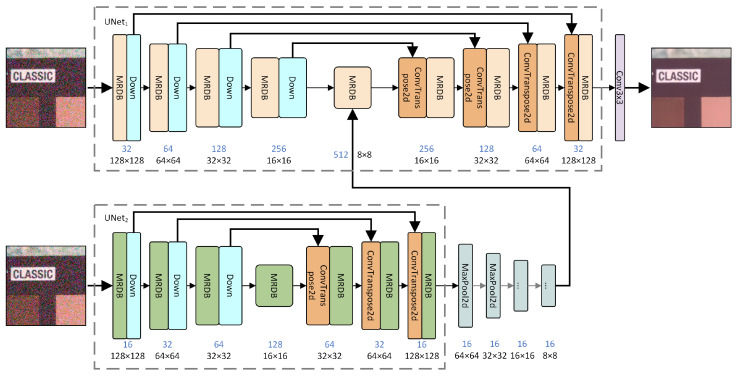
Network architecture of the denoiser.

The denoiser we designed, the Double-Layer UNet Denoising Network (DUNetD), uses a dual-UNet structure for image processing tasks. The architecture is shown in Figure 2. This dual UNet is an integrated learning-based serial application, enabling the two networks to extract and process image features at different levels. An input image $I\in R^{H\times W\times C}$ (where *H*, *W*, and *C* represent image height, width, and channels, respectively, with *C* being 3 for RGB images) is fed into both ${\mathrm{UNet}}_{1}$ and ${\mathrm{UNet}}_{2}$ simultaneously.

The encoder of the ${\mathrm{UNet}}_{1}$ network first processes the input image *I*, extracting features and reducing the spatial dimension through consecutive downsampling and Multi-Receptive Field Perception Denoise Module (MRDB) operations. This process yields feature maps $F_{1},F_{2},F_{3},$ and $F_{4}$, each with dimensions $R^{H_{i}\times W_{i}\times C}$, where $H_{i}$ and $W_{i}$ gradually decrease due to downsampling. Finally, the deep characteristics $F_{5}$ are derived from $F_{4}$ via the MRDB.

Meanwhile, the input image *I* is also fed into the encoder of ${\mathrm{UNet}}_{2}$, which undergoes similar downsampling and processing by the module ${\mathrm{MRDB}}_{2}$ to generate feature maps $f_{1},f_{2},$ and $f_{3}$, with $f_{4}$ subsequently derived from $f_{3}$. The decoder of ${\mathrm{UNet}}_{2}$ then restores the spatial dimensions of the image through upsampling and module processing, blending with the corresponding skip-connection feature maps to produce $f_{3}^{\prime },f_{2}^{\prime },$ and $f_{1}^{\prime }$.

Four consecutive convolutional layers are employed in the ${\mathrm{UNet}}_{2}$ output stage. Each layer has a kernel of 3×3, a stride of 2, and a padding of 1, progressively halving the image size. These layers reduce the size of $f_{1}^{\prime }$ step by step, yielding $O_{2}$.

The output $O_{2}$ of ${\mathrm{UNet}}_{2}$ is added to the deep feature $F_{5}$ of ${\mathrm{UNet}}_{1}$, forming the fused feature $F_{\mathrm{end}}$. This $F_{\mathrm{end}}$ guides the ${\mathrm{UNet}}_{1}$ decoder, which restores the image spatial dimensions via upsampling and MRDB′ module processing. The decoder merges with the corresponding skip-connection feature maps, generating $f_{4}^{\prime },f_{3}^{\prime },f_{2}^{\prime },$ and $f_{1}^{\prime }$. Finally, $f_{1}^{\prime }$ passes through the last processing layer (Conv3 × 3) to produce the final output image $O_{1}$, also sized $R^{H\times W\times 3}$.

The dual-UNet structure enables the two networks to extract and process image features at different levels. Feature fusion and skip connections combine their advantages for more efficient image processing. This design enhances the ability of the network to capture image details and improves processing accuracy and efficiency through collaboration between the two networks.

#### 3.2.1. Multi-Receptive Field Perception Denoise Module

**Figure 3:**
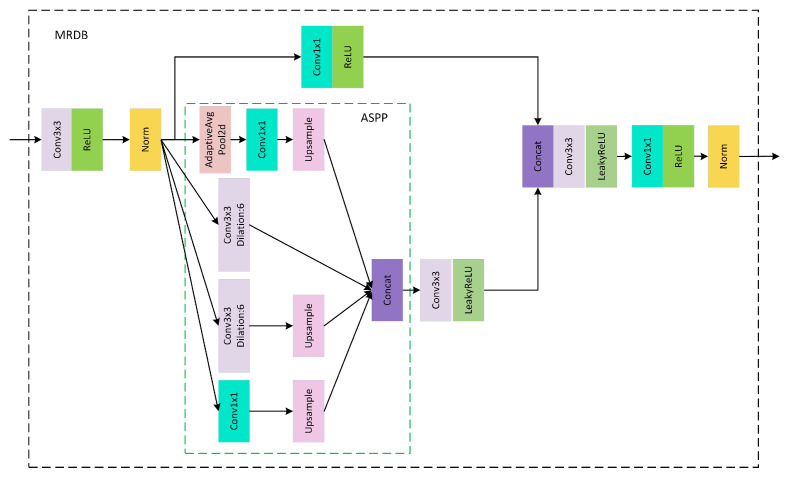
Multi-Receptive Field Perception Denoise Module.

We propose a Multi-Receptive Field Perception Denoise Module (MRDB) embedded in the dual-UNet structure of the image denoiser. The architecture is shown in Figure 3. The upper UNet and lower UNet utilize different ASPP modules to enhance multi-scale feature extraction, thereby improving the denoiser’s ability to capture features at various scales, as illustrated in the figure.

The core of the MRDB block is the ASPP submodule, which uses parallel convolutional kernels with different dilation rates to capture image info at multiple scales. During forward propagation, the input data are pre-activated and batch-normalized via a standard convolutional layer and then enter the ASPP submodule. In this module, the data pass through two convolutional layers with dilation rates of 3 and 6 and 4 and 8, respectively, depending on the ASPP version. Another path involves global average pooling, a 1×1 convolutional layer, and upsampling to match the output size of the feature extraction path. The outputs of these four paths are connected to form multi-scale features, which are then fused through convolutional layers and subjected to nonlinear transformations through activation functions. After another convolutional layer for further processing, the features undergo batch normalization and activation to produce the final output. Applying different ASPP modules in the dual UNet enhances the network’s feature extraction capability, improving noise removal and image quality restoration in denoising tasks.

#### 3.2.2. Enlarging the Receptive Field Module

In image denoising, obtaining multi-scale information is crucial for feature reconstruction. Although downsampling is common in networks, it can damage image structures and lose information in low-resolution cases, which is bad for feature reconstruction. To enlarge the receptive field, large-size convolutional kernels or large strides in pooling are often used; however, they both have high computational requirements and resolution loss issues.

Inspired by semantic segmentation models, we introduce an enlarged receptive field module into the dual-UNet structure. It can expand the receptive field and capture multi-scale information without increasing computation and sacrificing resolution. The ASPP module (Atrous Spatial Pyramid Pooling) [32], as a spatial pyramid pooling structure, is widely used in semantic segmentation [33] and other tasks. By applying convolutional kernels with different dilation rates in parallel, this module captures multi-scale contextual information, enabling the model to handle objects of varying sizes and improving its adaptability to complex scenes. Typically integrated into the decoder of deep Convolutional Neural Networks (CNNs), ASPP fuses multi-scale contextual information into feature maps, significantly enhancing segmentation accuracy. Importantly, it can achieve a large receptive field without reducing the size of the feature map [34].

Specifically, the ASPP structure employs a pooling layer and a 1×1 convolutional layer in the upper UNet, as well as two dilated convolutions with dilation rates of 3 and 6, respectively. The corresponding paddings are 3 and 6, with a stride of 1 and a kernel size 3×3 for both, ensuring consistent feature map dimensions for fusion. Subsequently, the LeakyReLU activation function applies a negative slope of 0.2. To obtain richer information on the feature, we first use a 1×1 convolution to expand the feature channel. We then concatenate the features obtained from different receptive fields in the fusion section and use a 3×3 convolution to compress the feature channels, forming a comprehensive feature representation.

Thus, the denoiser uses a dual-UNet structure. The module enlargement of the receptive field is in the upper UNet, while the lower UNet uses dilated convolutions with dilation rates of 4 and 8, corresponding paddings of 4 and 8, stride of 1, and 3×3 kernels, the same as in the upper UNet. The two UNets extract image features to different extents with different ASPP modules. This enables the denoiser to capture more details and multi-scale features, thereby better removing noise and restoring image quality in denoising tasks.

### 3.3. Generator U-Shaped Imitation Noise Generation Network

This architecture achieves effective simulation of complex data distribution through adversarial learning dynamics between the generator (U-shaped Imitation Noise Generation Network, UING) and the discriminator (Dual-channel Adversarial Discriminator, D2P). In recent years, researchers have proposed various image denoising methods based on GANs [35,36]. These methods have significantly improved noise reduction while preserving the image details. However, these methods may encounter pattern collapse and instability issues during the training process, leading to fluctuations in the quality of the generated images. To address these issues, this paper employs a simplified generator model that is matched to the denoiser to stabilize image quality. Moreover, the generated noisy images can further assist in training the denoiser, enhancing its ability to process noisy images.

**Figure 4:**
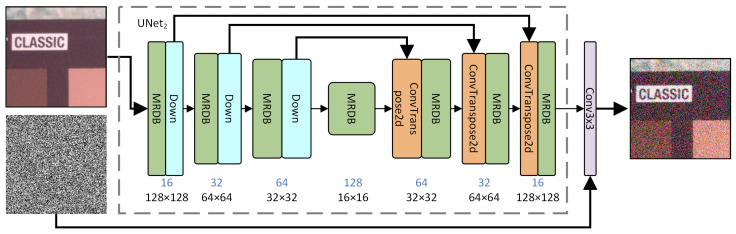
Network architecture of the generator.

In the generator, we adopt the ${\mathrm{UNet}}_{1}$ structure of the dual-UNet network used in the denoiser. The architecture is shown in Figure 4. It employs a single-input approach using ${\mathrm{UNet}}_{1}$ as the generator. The network takes a clean image $I\in R^{H\times W\times 3}$ as input (where *H*, *W*, and *C* represent the height, width, and channels of the image, respectively, with *C* being 3 for RGB images). By incorporating noise through network training, it is possible to generate noise simulation images similar to noisy images.

Due to its strong feature extraction capabilities and simple structure, the generator utilizes the upper UNet structure (${\mathrm{UNet}}_{1}$) of the dual-UNet network. This design choice enables the reuse of existing network architectures and parameters, thereby saving development time and computational resources. It also allows the application of established training and optimization techniques for efficient noise image generation. During training, the stability of the UNet architecture ensures quick convergence. Adjusting parameters and training strategies can control the intensity and type of noise generated. Moreover, the flexibility of ${\mathrm{UNet}}_{1}$ allows for customization to meet diverse application needs.

$$\begin{array}{c} min_{G}max_{D}V\left(D,G\right)=E_{x\;p_{data}\left(x\right)}\left[logD\left(x\right)\right]+E_{z\;p_{z}\left(z\right)}\left[log\left(1-D\left(G\left(z\right)\right)\right)\right] \end{array}$$

First, we define a prior input noise variable pz(z). A differentiable function G(z;θg) maps this noise to the data space, where θg represents its parameters. The goal is to learn the data-generating distribution pg. The discriminator D(x;θd), also a multi-layer perceptron, outputs a scalar indicating the probability that the input data *x* come from real data rather than pg.

During training, the discriminator *D* aims to maximize the probability of correctly distinguishing between real training samples and those generated by G. This means outputting a probability close to 1 for real data and close to 0 for data generated. At the same time, the generator *G* seeks to minimize log(1−D(G(z))) in order to fool the discriminator so that it cannot easily tell generated data from real data. This adversarial relationship forms a two-player minimax game, reflected through the value function V(G,D). As training progresses, D continuously improves its recognition ability, and G keeps improving the quality of the generated data. Eventually, this process drives the generator’s data distribution pg to gradually approach the real data distribution, achieving a dynamic equilibrium. The generator’s primary purpose is to create noise simulation images for data augmentation, especially when clean image data are limited. This significantly improves the generalization and robustness of the denoising model.

### 3.4. Discriminator Dual-Channel Adversarial Discriminator

**Figure 5:**
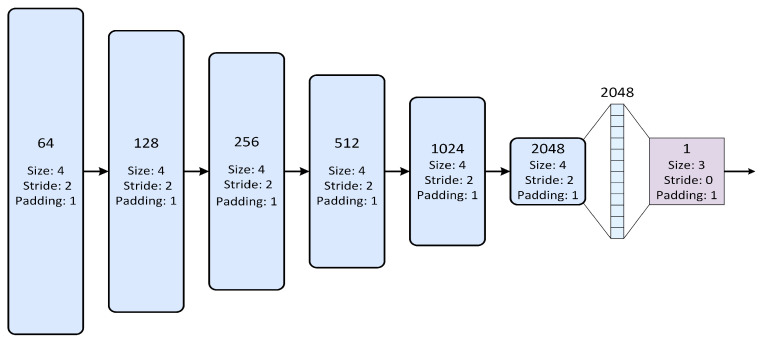
Network architecture of the discriminator.

The discriminator network [37] in this paper is a deep CNN-based structure. Its core consists of multiple consecutive convolutional layers, each of which is followed by a LeakyReLU activation function. The architecture is shown in Figure 5. The network, taking a clean RGB input image $I\in R^{H\times W\times C}$ (where *H*, *W*, and *C*, represent the height, width, and channels of the image, respectively, with *C* being 3 for RGB images), processes the input through a series of downsampling operations, and outputs a scalar value to judge the authenticity of the input image.

The discriminator network begins with a convolutional layer that maps the input image’s channels from Input to ndf using a 4×4 kernel, stride of 2, and padding of 1, thereby halving the spatial dimensions. Next, several convolutional layers progressively increase the channel count to ndf×2, ndf×4, ndf×8, ndf×16, all with the same kernel size, stride, and padding, followed by LeakyReLU for non-linearity. After these layers, the spatial size of the image is gradually reduced. Finally, the last convolutional layer compresses it to 1×1 with ndf×32 channels.

After convolution, the network flattens the feature maps into a vector and then maps it to a scalar output via a fully connected layer, indicating the discriminator’s judgment on the authenticity of the input image. The weights of all convolutional and fully connected layers are initialized with a normal distribution to ensure stability and optimal performance. This design enables the discriminator to effectively extract multi-scale features and enhance its ability to discern image authenticity through progressive downsampling.

### 3.5. Loss Function

**Figure 6:**
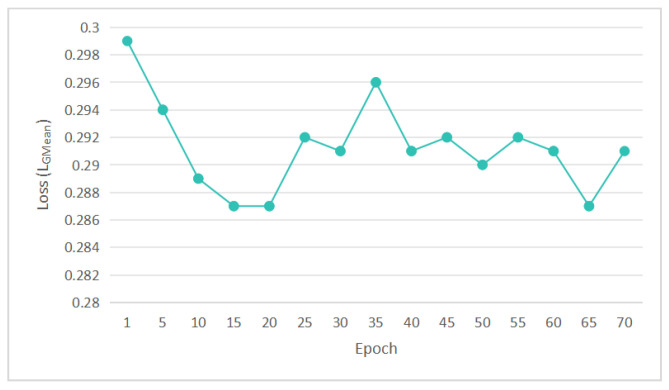
$L_{GMean}$ loss during training epochs.

The adversarial loss function trains the generator and discriminator by integrating the discrimination results of real data pairs, generated data pairs, and real clean images with simulated noisy images. Therefore, the generator can generate more realistic noisy images, and the discriminator can distinguish real from generated data more accurately. By adjusting the hyperparameter α, the impact of different data pairs during training is balanced, thereby optimizing the overall performance of the GAN model.

(1) $$\begin{array}{c} loss_{gan}=E_{\left(x,y\right)}\left[O\left(x,y\right)\right]-\alpha E_{\left(x^{\prime },y\right)}\left[O\left(x^{\prime },y\right)\right]-\left(1-\alpha \right)O_{\left(x,y^{\prime }\right)}\left[D\left(x,y^{\prime }\right)\right] \end{array}$$

where the parameters are defined as follows: *y* is the original noisy image serving as the input to the denoising model; $x^{\prime }$ represents the denoised image, which is the output of the model after processing the noisy image; $y^{\prime }$ is the simulated noisy image generated by adding noise to a clean image, used for model training; *x* stands for the original clean image, the ideal target and reference for denoising; O(x,y) is a real data pair consisting of a clean image *x* and its corresponding noisy image *y*, offering authentic data distribution for training; $O(x^{\prime },y)$ is a data pair from the denoiser, including the denoised image $x^{\prime }$ and the original noisy image *y*, reflecting the denoising effect; $O(x,y^{\prime })$ is a data pair obtained after the generator adds noise, based on the clean image *x* and the simulated noisy image $y^{\prime }$, used to train the generator in the GAN; E(x,y) denotes the expectation over real data pairs O(x,y), showing the overall distribution of real image data; $E(x^{\prime },y)$ is the expectation over generated data pairs $O(x^{\prime },y)$, assessing the denoiser’s overall performance on different noisy images; $E(x,y^{\prime })$ is the expectation over data pairs $O(x,y^{\prime })$, guiding the generator to produce more realistic noisy images. α is a weighting coefficient in the interval [0, 1] that balances the relative emphasis placed on the two adversarially generated data components in the loss: the denoised outputs and the synthetic noisy images. Specifically, as α approaches 1, the loss increasingly penalizes the discriminator’s response to denoised samples, requiring the denoiser to produce cleaner images that are indistinguishable from real clean data. In contrast, as α approaches 0, the loss shifts its focus toward penalizing the discriminator’s response to the generated noise, encouraging the generator to synthesize more realistic noisy images that faithfully approximate the accurate noise distribution. The $L_{GMean}$ loss during training epochs is shown in Figure 6.

(2) $$\begin{array}{c} L_{GMean}=E_{\left(x,y\right)}{∥GF\left(G\left(x\right)-GF\left(y-x\right)\right)∥}_{1} \end{array}$$

**Figure 7:**
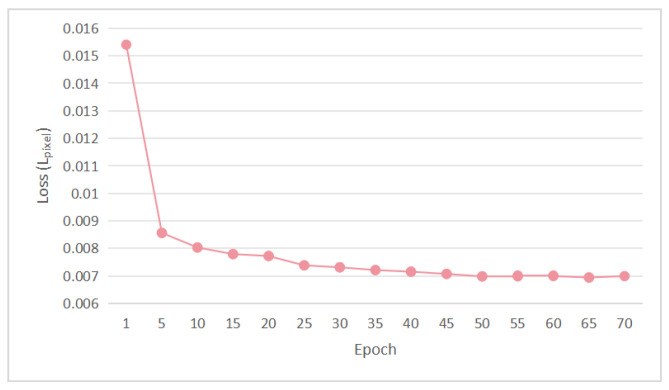
$L_{pixel}$ loss during training epochs.

The value of the feature-level loss with Gaussian filtering (GF) is absorbed into the term $L_{GMean}$ for the presentation of the output, where G(x) denotes the synthetic noise map produced by the generator. The function of the pixel-level difference loss ($L_{pixel}$) is to make the denoised image output by denoiser D as close as possible to the actual clean image at the pixel level, where D(y) is the output of the denoiser. The $L_{Gpixel}$ loss during training epochs is shown in Figure 7.

(3) $$\begin{array}{c} L_{pixel}=E_{\left(x,y\right)}∥\left(y-D\left(y\right)\right)-x){)∥}_{1} \end{array}$$

(4) $$\begin{array}{c} Loss=loss_{gan}+\gamma_{1}∥x^{\prime }{-x∥}_{1}+\gamma_{2}{∥GF\left(y^{\prime }-x\right)-GF\left(y-x\right)∥}_{1} \end{array}$$

The total loss function combines adversarial loss, pixel-level difference loss, and feature-level loss with Gaussian filtering (GF). It aims to make the generator’s noisy images closer to authentic images at multiple levels (pixel and feature). Hyperparameters $\gamma_{1}$ and $\gamma_{2}$ balance the impact of different loss terms to achieve better generation results.

$\gamma_{1}$ controls the weight of the loss at the pixel level $∥x^{\prime }{-x∥}_{1}$, which enforces the fidelity per pixel between the denoised output $x^{\prime }$ and the clean image of the ground truth *x*. A larger $\gamma_{1}$ emphasizes sharper pixel-wise reconstruction but may over-smooth fine textures. $\gamma_{2}$ controls the weight of the loss at the pixel level $∥GF\left(y^{\prime }-x\right)-GF\left(y-x\right){∥}_{1}$, which measures the structural similarity of noise patterns after Gaussian filtering. A larger $\gamma_{2}$ encourages the generator to synthesize noise whose low-frequency statistics (edges, smooth regions) closely match those of real noise, potentially at the expense of exact pixel values. According to a “grid search”, it was determined that $\gamma_{1}$ should be 1000 and $\gamma_{2}$ 10.

The balance between adversarial loss and pixel-level loss can be framed as a joint optimization of fidelity and perceptual authenticity. Adversarial loss ($Loss_{gan}$), driven by the discriminator’s game-theoretic mechanism, encourages the denoised output to align with the distribution of authentic clean images, thus enhancing visual naturalness. However, this mechanism does not offer an explicit constraint on pixel-wise consistency. To compensate, the loss of L1 at the pixel-level intervenes with a dominant weight $\gamma_{1}$ of 1000, rigidly correcting any detail drift that the adversarial loss may induce ($\gamma_{1}∥x^{\prime }-x{∥}_{1}+\gamma_{2}∥$). This strategy effectively reformulates the optimization objective into a constrained minimax problem. By ensuring that the discriminator cannot distinguish between authentic and generated images, we further restrict the solution space to those outputs that exhibit strict pixel-wise overlap with the ground-truth image. This approach preserves both reconstruction accuracy and perceptual quality.

## 4. Experiment and Result Analysis

### 4.1. Measurement Standards

In our research, the overall quality of the denoised images was evaluated using the Peak Signal-to-Noise Ratio (PSNR) [38] and the Structural Similarity (SSIM) [39].

(5) $$\begin{array}{c} PSNR=10lg\{\frac{Max^{2}\left[G_{t}\left(i,j\right)\right]}{\frac{1}{HW}\sum_{i=1}^{H}\sum_{j=1}^{W}{\left[G_{t}\left(i,j\right)-N\left(i,j\right)\right]}^{2}}\} \end{array}$$

(6) $$\begin{array}{c} SSIM=\frac{\left(2u_{1}u_{2}+c_{1}\right)\left(2\sigma_{1,2}+c_{2}\right)}{\left(u_{1}^{2}+u_{2}^{2}+c_{1}\right)\left(\sigma_{1}^{2}+\sigma_{2}^{2}+c_{2}\right)} \end{array}$$

Here, $G_{t}\left(i,j\right)$ is the pixel value of the original noise-free image at position (i,j), and N(i,j) is the pixel value of the denoised image at position (i,j). *H* and *W* represent the height and width of the image. The mean of $G_{t}\left(i,j\right)$ and N(i,j) are represented by $u_{1}$ and $u_{2}$. The variances of $G_{t}\left(i,j\right)$ and N(i,j) are indicated by $\sigma_{1}$ and $\sigma_{2}$. The covariance between $G_{t}\left(i,j\right)$ and N(i,j) is represented by $\sigma_{1,2}$. Constants c1 = 0.01 and c2 = 0.02 are introduced to maintain system stability.

Let $G_{e}$ be the ground-truth edge map, $D_{e}$ be the detected edge map, and *k* be a constant controlling distance influence. Let *d* be the Euclidean distance between the ground truth and the detected edges. $|G_{e}|$ and $|D_{e}|$ denote the pixel counts of these maps. p indexes each pixel position in the image, iterating through all pixels during FOM computation.

The quality of edge detection in the images was assessed using a metric called the Figure of Merit (FOM). It measures the location and magnitude of detected edges to ensure precision in edge detection.

(7) $$\begin{array}{c} FOM=\frac{1}{Max\left(\right|G_{e}\left|\right|D_{e}\left|\right)}\sum_{p=1}^{HW}\frac{1}{1+k.d_{G_{e}}^{2}\left(p\right)} \end{array}$$

By calculating the absolute error between the denoised image and the real image in each pixel and taking the average, the quality of the denoising effect can be measured.

(8) $$\begin{array}{c} MAE=\frac{1}{HW}\sum_{i=1}^{H}\sum_{j=1}^{W}\left|G_{t}\left(i,j\right)-N\left(i,j\right)\right| \end{array}$$

**Figure 8:**
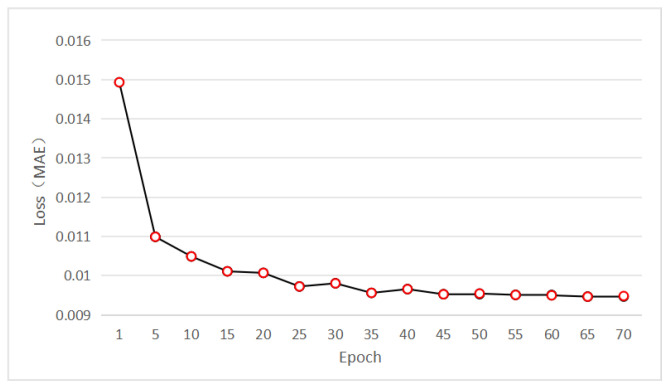
MAE loss during training epochs.

$G_{t}\left(i,j\right)$ is the pixel value of the original noise-free image at position (i,j), and N(i,j) is the pixel value of the denoised image at position (i,j). The MAE loss during training epochs is shown in Figure 8.

### 4.2. Experimental Dataset

In this experiment, three datasets with real-noise images were used: SIDD [40], DND [41], and PolyU [42]. They are invaluable for research on denoising algorithms.

The Smartphone Image Denoising Dataset (SIDD) contains 30,000 noisy images in 10 scenes. These images were captured using five representative smartphone cameras under different lighting conditions. Moreover, the SIDD provides ground-truth images corresponding to the noisy ones, laying a solid data foundation for denoising algorithm research.

The Darmstadt Noise Dataset (DND) comprises 50 images captured by four consumer cameras with different sensor sizes. This ensures data diversity and authenticity. For each pair of images, the reference image (ground truth) was shot at the base ISO, while the noisy image was shot at a higher ISO with adjusted exposure. The reference images underwent careful post-processing, including minor camera shift correction, linear intensity scaling, and removal of low-frequency bias to ensure their accuracy as a benchmark. However, since the noise-free images in this dataset are not publicly available, all images were used for testing only.

The PolyU dataset captured 40 scenes and computed the average of these images to create a near-true “ground truth” (a noise-free or low-noise reference). This leveraged the randomness of noise, using multiple samples and averaging to approximate absolute pixel values and reduce noise. Since the PolyU dataset includes noisy images and noise-free or low-noise references in the real world, it was also used as a test set for comparative analysis.

The training images from the SIDD-Medium dataset were used as the training data in this paper’s experiment. Given the large size of the SIDD training images, 300 random 256 × 256 patches were cropped from each original image pair, yielding 96,000 patches. The model was then trained on these smaller patches. For testing, the DND dataset, the testing images from the SIDD dataset, and the PolyU dataset were used, and all tests were performed on RGB images.

### 4.3. Comparison Method

#### 4.3.1. Comparison of Key Methods

ID-MSE-WGAN is suitable for environments with limited hardware resources [12], which restricts its application in resource-constrained settings. The complexity of its network architecture restricts its capability for feature extraction and fusion. Additionally, its lack of image detail processing results in a gap between the actual denoising effects and theoretical expectations.

In contrast, our approach introduces an early-stopping mechanism. This effectively controls training time, prevents overfitting, and enhances the model’s generalization ability. Our design also includes a specialized module for processing image details. This module maintains high computational efficiency while significantly improving feature extraction and image processing performance.

The Adversarial Gaussian Denoiser Network (AGDN) combines adversarial loss and L1 loss for pixel reconstruction [9], thus optimizing the denoising network. Adversarial loss improves image clarity and sharpness, while L1 loss ensures that the generated images are closely aligned with the target images. However, dynamically adjusting the weights of these two losses across different noise levels and image content for optimal denoising remains a challenge. Moreover, AGDN’s complexity may lead to slow inference speeds in practical applications, making it unsuitable for real-time, demanding scenarios.

Our method uses the MAE loss function. It enhances the quality of the generator’s image generation, supervises the denoiser’s denoising performance, and maintains the overall model’s efficiency and stability. The dual-input design of the dual-UNet network may extend training time. However, the similarity of the top-down architecture does not slow down the inference speed in practical applications. Instead, it further enhances the model’s adaptability to complex scenarios.

CS-PCN employs a three-stage serial–parallel hybrid architecture. The first two stages comprise the cascaded Context Mining Twin Sub-network (CM2S), which is composed of an MLFP dilated convolution, an AED encoder–decoder, and an MCAC multi-head attention controller. These stages are responsible for extracting multi-scale semantic information. The third stage is a parallel Spatial Synthesis Sub-network (3S) that refines local details via cascaded dual-attention blocks. The two feature streams are finally fused by adding elements in a way that is elemental. This staged refinement strategy achieves a compromise between noise suppression and detail preservation. Nevertheless, the fixed serial–parallel topology of CS-PCN limits its adaptability to complex real-world noise; the absence of an adversarial mechanism hinders learning of unknown noise distributions; the effective receptive field is still bounded by network depth; and robustness degrades under training–testing domain shifts.

In contrast, our denoiser generalizes to unseen noise distributions without additional data augmentation. The dual-UNet structure enlarges the receptive field through cascaded-parallel synergy, performing hierarchical feature fusion that jointly ensures statistical fidelity and texture recovery. This design directly enhances robustness under complex and unknown noise scenarios.

Trident GAN introduces the Trident Block for feature extraction [13], which includes the Local Detail Enhancement Block (LDEB), Lightweight Channel Attention Block (LCAB), and Spatial Interaction Attention Block (SIAB). These modules extract local detail features, channel features, and spatial features, respectively, with feature aggregation achieved through a Multi-Feature Fusion (MFF) module [13]. This design improves feature utilization efficiency but has room for improvement in multi-scale feature extraction. Our approach employs the Multi-Receptive Field Perception Denoise Module (MRDB) embedded in a dual-UNet structure. The MRDB uses dilated convolutions with different rates to capture multi-scale features in parallel. This increases feature extraction efficiency and leverages the skip connections in UNet for more effective feature fusion. The dual-UNet network’s similar structure and dual-input design enable better feature fusion for different detail-feature tasks, further enhancing the model’s adaptability to complex scenes.

Trident GAN is suitable for environments with limited hardware resources and offers short testing times, making it ideal for scenarios that demand real-time performance. However, its feature extraction and fusion capabilities are constrained by the complexity of its network structure. In contrast, our proposed design is designed for medium-sized datasets. By incorporating an early-stopping mechanism, our approach effectively controls training time, preventing overfitting and enhancing the model’s generalization ability. This design maintains high computational efficiency and significantly improves feature extraction and image processing performance. The results of comparison are shown in Table 1.

#### 4.3.2. Original Design Proposal

In exploring the concatenation design for the denoiser’s dual-U structure, various methods were initially tried. However, experiments revealed that these initial approaches yielded poor denoising results and underperformed in metric evaluations.

When connecting ${\mathrm{UNet}}_{1}$ and ${\mathrm{UNet}}_{2}$ sequentially and abandoning the dual-channel input in favor of a single input at the ${\mathrm{UNet}}_{1}$ entrance, with processing through both networks leading to an output at the ${\mathrm{UNet}}_{2}$ exit, the resulting structure resembled the letter “W” and was termed WNet. However, experimental results indicated that WNet performed poorly in image denoising. This was mainly attributed to insufficient feature fusion and gradient vanishing. In the original dual-UNet structure, feature fusion effectively combined the strengths of both networks, thereby enhancing the denoising effect. In the sequential structure, this fusion was compromised, as ${\mathrm{UNet}}_{2}$ could only process ${\mathrm{UNet}}_{1}$’s final output without access to its intermediate features. Moreover, the increased network depth from sequential connection may intensify gradient decay during backpropagation, negatively impacting training effectiveness and convergence speed.

Connecting the bottoms of ${\mathrm{UNet}}_{1}$ and ${\mathrm{UNet}}_{2}$ while maintaining dual-channel inputs from both entrances and producing separate outputs before fusing them into a single result created a structure resembling the letter “X”, hence the network was named XNet. Experiments showed that XNet’s denoising performance was even worse than that of WNet. This could be due to gradient conflicts or information interference during training after connecting the two UNet networks at the bottom. Such issues likely arose because the connected bottom layers disrupted information flow, leading to insufficient and interfering feature fusion. Consequently, the networks failed to fully utilize their feature extraction capabilities, negatively impacting the overall denoising effect.

During the dual-U design, a triple-U structure (U3Net) was proposed by adding a ${\mathrm{UNet}}_{3}$ connected to the bottom of ${\mathrm{UNet}}_{2}$. U3Net had three inputs and one output. Although performing better than WNet and XNet, U3Net’s training took longer, and its final effect still lagged behind the proposed dual-U structure. While the UNet-based network could effectively utilize multi-level features and accelerate convergence, this was optimal at a certain depth. U3Net, though extracting diverse features, suffered from high complexity, longer training time, and inefficient feature fusion due to significant feature differences at connection points, making it less efficient than the final dual-U structure. The results of different design structures are shown in Table 2.

#### 4.3.3. Computational Efficiency

In terms of parameters and FLOPs, the generator utilizes a 32-channel UNet backbone with approximately 7.8 M parameters and 34.2 GFLOPs per forward pass. The denoiser consists of two parallel UNet branches, which doubles the parameter count to 15.6 M and the computation to 68.9 GFLOPs. The discriminator is a 64-channel PatchGAN, comprising only 4.2 M parameters and 9.1 GFLOPs—the lightest component. In aggregate, the entire framework contains 27.6 M parameters and consumes 112.2 GFLOPs, well within the capability envelope of contemporary edge-side hardware. Training time was measured on 96k 64×64 patches from the SIDD dataset using a single V100S GPU (NVIDIA, Santa Clara, CA, USA). One epoch required approximately 9 min for the generator, 18 min for the denoiser, and 5 min for the discriminator; the complete adversarial iteration took a total of 32 min. With early stopping, total training converged within 46 h—comparable to the 40 h of a single-path UNet baseline—demonstrating that the dual-path design yielded substantial performance gains without incurring excessive training overhead. We performed inference delay testing with “batch = 1” on 100 512×512 images. Average runtimes were 18 ms for the generator, 38 ms for the denoiser, and 12 ms for the discriminator, resulting in an end-to-end latency of 55 ms, which is below the 100 ms threshold typically required for real-time applications. This confirms an effective balance between performance and efficiency. The calculation results are shown in Table 3.

### 4.4. Comparative Experiments

In this experiment, we built neural network models using the PyTorch framework. The hardware setup included a Tesla V100S-PCIE-32 GB GPU with 32 GB of memory, an Intel(R) Xeon(R) Gold 5220R CPU, and 32 GB of system RAM. We set the initial number of channels to 32 for the denoiser and generator and 64 for the discriminator. The initial learning rates for the denoiser and generator were $1\times 10^{-4}$, and for the discriminator, it was $2\times 10^{-4}$. All model parameters were updated using the Adam optimizer. To prevent overfitting, we implemented an early-stopping mechanism. Training would stop automatically when the validation loss plateaued, thereby enhancing generalization and avoiding the waste of computational resources. The details of the environment configuration are shown in Table 4.

**Figure 9:**
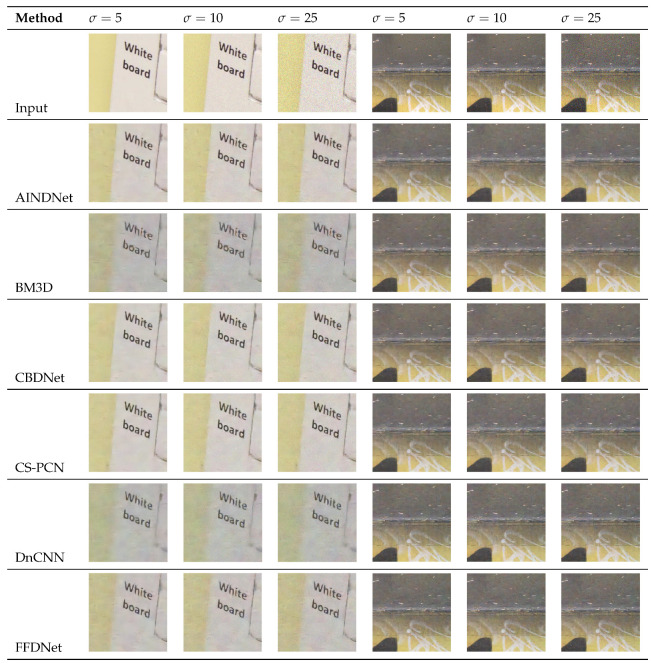

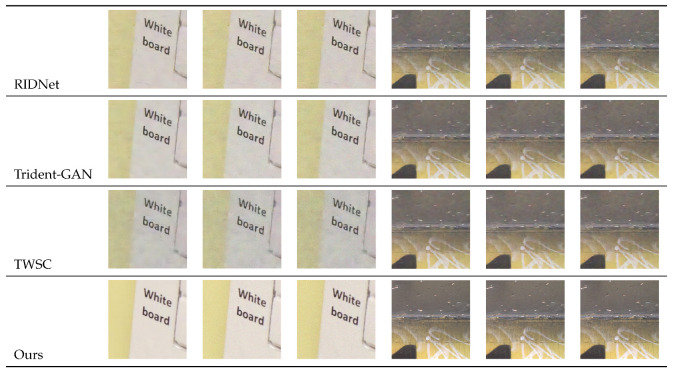
Comparison of denoising results on PolyU (**left**) and DND (**right**) datasets at noise levels σ of 5, 10, and 25.

Our experiment compared the denoising performance of the proposed network using test images from the DND dataset, SIDD Medium dataset, and PolyU dataset. The model was trained for 70 epochs, which took approximately 46 h. Table 5 and Table 6 summarize the quantitative comparisons of different algorithms in terms of PSNR and SSIM metrics. The PSNR for the SIDD-Medium dataset was tested uniformly on the RGB channels, while that of the DND dataset was obtained through the official system. The PolyU dataset contains only noisy images, so this paper provides a visual comparison. Nine denoising algorithms (AINDNet [43], BM3D [8], CBDNet [44], CS-PCN [45], DnCNN [10], FFDNet [46], RIDNet [47], Trident GAN [13], and TWSC [48]) were used for comparison. The results of comparative experiment are shown in Table 5 and Table 6, Figure 9.

### 4.5. Ablation Studies

To verify the effectiveness of the design, we conducted a series of ablation studies on three key components of the denoiser: the dual U structure, the MRDB module, and the ASPP module (part of MRDB). The results are summarized in Table 7.

- Experimental settings:
  1. Dataset: SIDD, consistent with previous comparative experiments.
  2. Parameters: All experimental parameters were strictly set to the same values for accuracy and reproducibility of the results.
- Experimental operation:
  1. We removed the lower U-shaped structure (${\mathrm{UNet}}_{2}$), retaining only the upper U-shaped structure (${\mathrm{UNet}}_{1}$).
  2. We removed the MRDB module. When the MRDB module was removed, the ASPP module was also excluded due to its inherent association with it.
  3. We removed the ASPP module.
- Results:
  1. Compared to having no modules, the PSNR was improved by 2.83 dB, 2.89 dB, and 2.39 dB.
  2. Compared to having no modules, the SSIM was improved 4.14%, 5.4%, and 5.31%.
  3. Impact of removing all modules: the PSNR decreased by 2.96 dB and the SSIM by 0.056%, indicating a significant performance drop.

The dual UNet removed the lower UNet (${\mathrm{UNet}}_{2}$) and retained only the upper UNet (${\mathrm{UNet}}_{1}$), resulting in a significant drop in PSNR and SSIM. This is because the dual UNet extracted and fused multi-scale features at different levels through the collaboration of the two networks. With ${\mathrm{UNet}}_{2}$ removed, the receptive field shrunk, feature fusion was lost, and the ability to capture complex image details diminished. In addition, the collaborative effect of the dual UNet was weakened, leaving the decoder with insufficient fused features for guidance and thereby reducing the image restoration quality. Moreover, the skip connections in ${\mathrm{UNet}}_{2}$ conveyed low-level features, and their removal caused the decoder to lose details and spatial information, thereby impacting the image’s structural similarity (SSIM). The dual UNet was highly adaptable to complex scenes, and the removal of ${\mathrm{UNet}}_{2}$ weakened its noise suppression ability, resulting in a decrease in PSNR and SSIM. The dual UNet enhanced image processing results through collaborative work and multi-scale feature fusion. Removing ${\mathrm{UNet}}_{2}$ eliminated these advantages, resulting in a decrease in the indicators.

The ASPP module captures multi-scale contextual information through dilated convolutions with varying rates, thereby enhancing the model’s ability to capture features of different scales. This helps to handle the details and noise in images more effectively. Removing ASPP weakened the network’s multi-scale feature extraction, reduced the receptive field, and diminished feature fusion, resulting in a slight drop in denoising performance. ASPP concatenates and fuses features from different receptive fields to form a comprehensive representation, enriching feature information and boosting the model’s adaptability and robustness. ASPP optimizes computational efficiency without significantly increasing computation compared to large-kernel convolutions or large-stride pooling. In experiments, using ASPP led to slight improvements in PSNR and SSIM, indicating its positive role in detail restoration and structure preservation and markedly enhancing the model’s performance in image denoising.

As shown in Table 7, the dual-U structure is crucial to improve the PSNR and SSIM of the network. The MRDB module significantly boosts the network’s SSIM performance. The ASPP module further optimizes the SSIM. These three modules operate in tandem within the network architecture, effectively enhancing denoising performance. Their combined use maximizes denoising results, confirming the rationality and efficiency of our design.

### 4.6. Superparameter Experiment and Result Analysis

**Figure 10:**
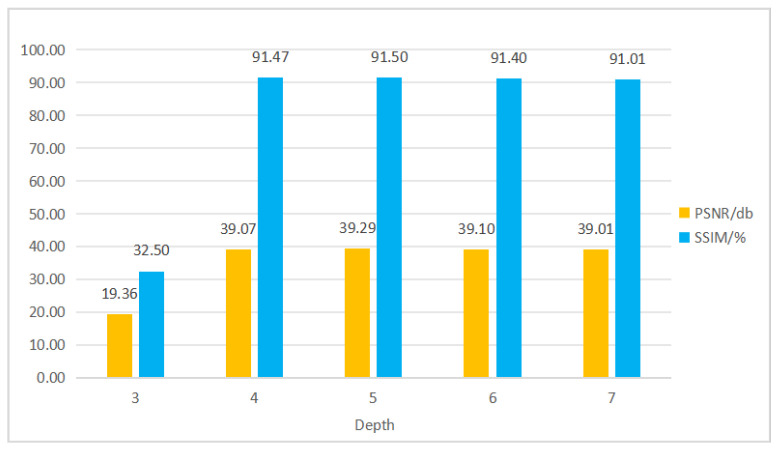
Comparison of the impact of network depth.

When fine-tuning the denoiser’s hyperparameters, network depth and the number of training epochs are key factors that significantly impact denoising performance. Experimental data suggested that changes in network depth and the number of training epochs affected the PSNR and SSIM, two metrics commonly used for image quality assessment. The results of experiment are shown in Figure 10.

**Figure 11:**
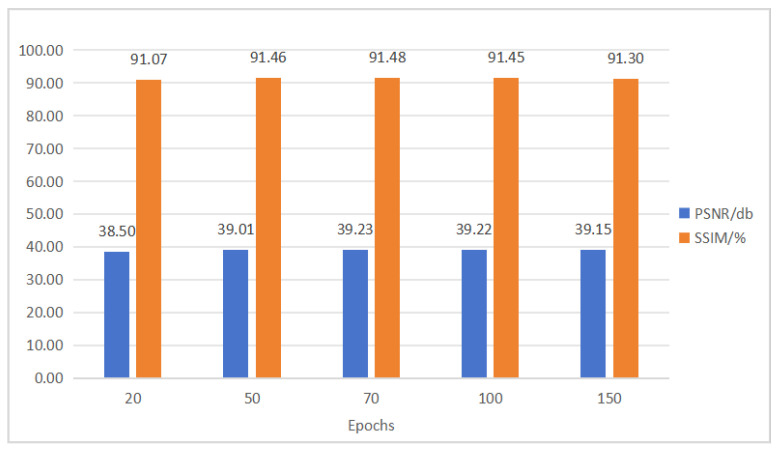
Comparison of the impact of training epochs.

As the number of training epochs increased, the denoiser’s performance initially improved, then stabilized, and could even decline slightly. At 20 epochs, the PSNR was 38.5 dB, and the SSIM was 91.07%, indicating insufficient learning of image features and noise patterns. Performance improved and stabilized at 50, 70, and 100 epochs, with peak PSNR and SSIM at 39.29 dB and 91.5%, respectively, at 70 epochs. This shows that proper training duration optimizes parameters and enhances denoising. However, at 150 epochs, PSNR and SSIM slightly dropped, possibly due to overfitting and reduced generalization. Additionally, excessive training may trap the network in local optima. The results of experiment are shown in Figure 11.

Therefore, both network depth and the number of training epochs impact the denoiser’s performance. The appropriate depth and epochs enable the denoiser to balance the PSNR and SSIM well. However, excessive depth or training time may degrade performance. This phenomenon is attributed to the unique decoder and skip connections of the U-shaped network, which enable rapid convergence, particularly on medium-sized datasets. Therefore, in practical applications, network depth and training epochs should be chosen based on specific task requirements and data characteristics to optimize the denoiser’s overall performance.

## 5. Conclusions

We introduced an advanced image denoising framework comprising three parts, denoising, generator, and discriminator, which combined the dual-path adversarial mechanism with an enhanced dual-UNet encoder–decoder structure. Adversarial training enhanced the model’s adaptability to various types of noise, while the dual-UNet architecture effectively extracted multi-scale features and fused dense features, resulting in significant improvements in noise removal and detail restoration. The experiment verified the superior performance of the proposed model, effectively overcoming the limitations of traditional methods and providing a robust solution for image denoising.

The proposed dual-path adversarial denoising network demonstrated robust performance across diverse noise types; however, its effectiveness was contingent upon the characteristics of the noise and the parameter configurations. While the model excelled in handling common noise patterns (e.g., Gaussian, salt-and-pepper noise), its stability may degrade with structured or non-stationary noise, such as low-frequency Perlin noise. Perlin noise, which introduces spatial coherence and gradient variations, challenges the model’s reliance on local receptive fields, potentially leading to residual artifacts or over-smoothing in homogeneous regions. Parameter sensitivity further influences performance. Variations in noise intensity or distribution (e.g., altering Gaussian standard deviation or spatial correlation) can disrupt the adversarial equilibrium. For instance, aggressive noise suppression may erode fine textures, while conservative settings might retain noise. The dual UNet with fixed dilation rates in ASPP modules may also limit adaptability to noise scales outside the trained range. Future work could explore dynamic dilation mechanisms or noise-specific attention modules to enhance generalization. These limitations highlight the trade-off between generality and specialization, indicating the need for noise-aware training protocols or hybrid methods to cope with complex real-world scenarios, which is precisely the problem we need to address in the future.

## Figures and Tables

**Table 1:** Results of the comparison of key design structures on the SIDD dataset.

Method	PSNR/dB	SSIM/%	FOM	TT/s
ID-MSE-WGAN	29.28	37.14	0.538	-
ADGN	26.01	87.09	0.7251	0.064
CS-PCN	38.53	90.56	0.9119	0.059
Trident GAN	38.40	90.40	0.9120	0.047
Ours	39.29	91.50	0.9121	0.051

**Table 2:** The results of different design structures ion the SIDD dataset.

Method	PSNR/dB	SSIM/%
WNet	23.67	33.38
XNet	12.87	37.37
U3Net	37.72	89.28

**Table 3:** Calculation results.

Module	Parameter (M)	FLOPs (G)	Single Epoch Training (min)	Single Image Reasoning (ms)
Generator (32 channel)	≈ 7.8	34.2	9	18
Denoiser	≈15.6	68.9	18	38
Discriminator	≈4.2	9.1	5	12
Overall	27.6	112.2	32	55

**Table 4:** Environment Configuration.

Configuration	Parameter
Operating system	Ubuntu 20.04.6 LTS
GPU	Tesla V100S-PCIE-32 GB
CPU	Intel(R) Xeon(R) Gold 5220R CPU @ 2.20 GHz
Deep learning framework	PyTorch 1.3.1
Python version	3.7.4

**Table 5:** The results on the SIDD dataset.

Method	PSNR/dB			SSIM/%			FOM/%		
	σ=5	σ=10	σ=25	σ=5	σ=10	σ=25	σ=5	σ=10	σ=25
DnCNN	23.66	24.67	24.52	58.30	57.37	62.57	62.57	62.58	62.77
BM3D	25.65	25.71	25.67	68.50	68.61	68.71	68.50	68.61	68.71
TWSC	26.02	27.95	27.82	62.02	61.95	63.82	67.02	66.95	67.82
FFDNet	33.07	33.28	33.48	62.25	62.30	62.50	72.67	72.75	72.52
CBDNet	30.77	35.71	38.02	76.37	86.30	86.80	77.25	77.16	77.34
RIDNet	32.25	32.31	32.36	81.16	80.28	80.97	79.27	79.65	79.85
AINDNet	32.22	32.32	32.41	80.21	80.15	81.03	79.11	80.01	80.21
Trident GAN	38.10	38.40	38.69	90.10	90.40	90.19	89.10	91.20	91.19
CS-PCN	38.34	38.53	38.73	90.22	90.56	90.89	89.98	91.19	91.21
Ours	38.91	39.29	39.10	91.13	91.50	92.01	91.03	91.21	91.30

**Table 6:** The results on the DND dataset.

Method	PSNR/dB			SSIM/%			FOM/%		
	σ=5	σ=10	σ=25	σ=5	σ=10	σ=25	σ=5	σ=10	σ=25
DnCNN	32.47	32.43	32.75	79.13	79.11	79.07	68.57	68.51	67.77
BM3D	34.51	33.20	34.89	85.15	85.10	85.24	69.70	69.61	69.68
TWSC	34.12	33.15	34.82	84.02	84.95	84.82	70.01	71.32	71.82
FFDNet	34.25	34.45	34.50	84.25	84.60	84.50	77.55	77.99	78.50
CBDNet	38.05	37.71	38.02	84.07	84.21	84.02	78.77	78.71	78.72
RIDNet	38.25	38.21	38.26	85.16	85.28	85.05	79.76	80.02	80.15
AINDNet	38.37	38.41	38.39	85.21	85.05	85.15	80.21	80.17	80.35
Trident GAN	38.90	39.10	39.19	93.10	93.40	93.19	93.10	93.19	93.20
CS-PCN	39.01	39.02	39.09	93.11	93.30	93.89	93.11	93.30	93.89
Ours	39.23	39.48	39.31	94.03	94.48	94.52	94.01	94.22	94.36

**Table 7:** The results of ablation studies on the SIDD dataset.

Method	PSNR/dB	SSIM/%
None	36.33 ± 0.10	85.91 ± 0.11
Dual UNet	39.16 ± 0.15	90.05 ± 0.17
Dual UNet without ASPP	39.22 ± 0.12	91.31 ± 0.15
MRDB	38.72 ± 0.11	91.22 ± 0.14
Ours	39.29 ± 0.05	91.50 ± 0.09

## Data Availability

The data presented in this study are openly available in SIDD at DOI:10.1109/CVPR.2018.00182, reference number [40]; DND at DOI:10.1109/CVPR.2017.294, reference number [41]; PolyU at DOI:10.48550/arXiv.1804.02603, reference number [42].
